# Indoor Particulate Matters, Microbial Count Assessments, and Wheezing Symptoms among Toddlers in Urban Day Care Centers in the District of Seremban, Malaysia

**DOI:** 10.5334/aogh.2425

**Published:** 2019-01-22

**Authors:** Raihan Khamal, Zaleha Md Isa, Rosnah Sutan, Nor Mohd Razif Noraini, Hasanain Faisal Ghazi

**Affiliations:** 1 Department of Community Health, Faculty of Medicine, Universiti Kebangsaan, Bangi, MY; 2 Industrial Hygiene Division, National Institute of Occupational Safety and Health (NIOSH), Bangi, MY; 3 Community Medicine Unit, International Medical School, Management and Science University, Shah Alam, Selangor, MY

## Abstract

**Introduction:**

Indoor air quality in day care centers (DCCs) is an emerging research topic nowadays. Indoor air pollutants such as particulate matter (PM) and microbes have been linked to respiratory health effects in children, particularly asthma-related symptoms such as night coughs and wheezing due to early exposure to indoor air contaminants.

**Objective:**

The aim of this study was to determine the association between wheezing symptoms among toddlers attending DCCs and indoor particulate matter, PM10, PM2.5, and microbial count level in urban DCCs in the District of Seremban, Malaysia.

**Methods:**

Data collection was carried out at 10 DCCs located in the urban area of Seremban. Modified validated questionnaires were distributed to parents to obtain their children’s health symptoms. The parameters measured were indoor PM2.5, PM10, carbon monoxide, total bacteria count, total fungus count, temperature, air velocity, and relative humidity using the National Institute for Occupational Safety and Health analytical method.

**Results:**

All 10 DCCs investigated had at least one indoor air quality parameter exceeding the acceptable level of standard guidelines. The prevalence of toddlers having wheezing symptoms was 18.9%. There was a significant different in mean concentration of PM2.5 and total bacteria count between those with and those without wheezing symptoms (*P* = 0.02, *P* = 0.006).

**Conclusions:**

Urban DCCs are exposed to many air pollutants that may enter their buildings from various adjacent sources. The particle concentrations and presence of microbes in DCCs might increase the risk of exposed children for respiratory diseases, particularly asthma, in their later life.

## Introduction

The quality of indoor air has become an increasing public health concern because most people spend 70%–90% of their time indoors, particularly children. The complex mixture of air pollutants, including particulates, gaseous materials, and microorganisms, affects young children, who are more sensitive to indoor air quality (IAQ) due to their immature immune systems, greater inhaled breath per unit mass, breathing zone nearer to the ground, and rapid growth [1]. Most Malaysian parents who are both working send children as early as three months old to day care centers (DCCs) established by the government or private agencies. They may spend eight hours per day, five days per week in DCCs, and some even spend over 10 hours per day, depending on the time they arrive at and leave the center [2]. Hence, it is substantially important that good IAQ be established in DCCs.

Early exposure to indoor air pollutants among children attending DCCs might increase the risk of allergies and asthma [3,4]. Although no definitive proof exists, day care attendance was found to be a risk factor for asthmatic symptoms—usually wheezing and night cough, especially among children less than five years old [5,6]. In a recent study, indoor particulate matter (PM) with aerodynamic diameter of 2.5 mm (PM2.5) were found to be associated with wheezing and night cough in asthmatic children [7]. Young children who are exposed to ultrafine particles have increased risk of respiratory infection, especially those who have asthmatic symptoms [8]. In addition to indoor particles, bioaerosols such as airborne bacteria and fungus are also associated with respiratory symptoms among children. High exposure to toxic-irritant complexes produced by fungi and bacteria are associated with increased occurrence of symptoms suggestive of asthma in young children [9,10]. Risk factors in DCCs such as inadequate ventilation, various indoor materials, high occupancy, and humidity [11] expose children to various levels of indoor air pollutants. This study aimed to determine the association between indoor particulates and airborne bioaerosols at Malaysian DCCs in urban areas and asthmatic symptoms among toddlers one to four years of age.

## Materials and Methods

### Study Design and Study Location

**Figure 1:**
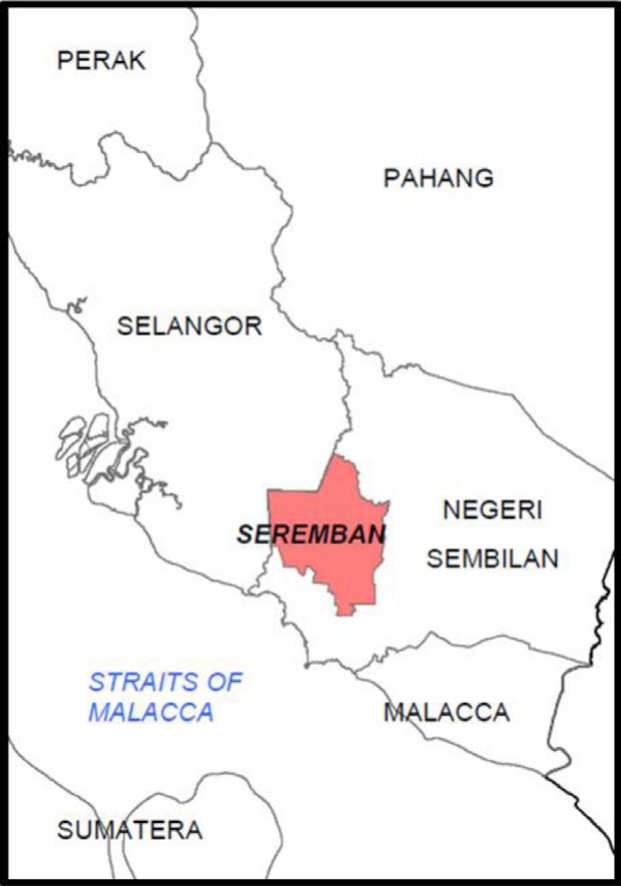
Location of Seremban District, Negeri Sembilan. (Adapted from Shamsuddin and Yaakup [14]).

This cross-sectional study was carried out in the District of Seremban in Negeri Sembilan, Malaysia. Seremban is an urban and industrialized area [12] located in the south of the country, about 54 km (33 miles) from Kuala Lumpur, the capital city of Malaysia (Figure 1) [13].

### Study Population/Sampling

A total of 40 out of 80 registered DCCs in the district of Seremban agreed to participate in this study. The list of DCCs was obtained from the Welfare Departments of the District of Seremban. The owners of the DCCs were contacted via phone and told about the study. Written informed consent was obtained from the participants. Of 40 DCCs, only 10 had mechanical ventilation (air conditioning); these were chosen as controls.

The respondents from the 10 DCCs were selected by a simple random sampling method with several inclusion criteria, namely children aged one to four years who were healthy, free from any respiratory illness, and had attended the same DCC for at least six months. The names of the children were obtained from the teachers. A set of questionnaires was given to parents to obtain information regarding respondent characteristics, and a set of questionnaires was given to the owners of the DCCs to get information regarding the characteristics of the associated buildings. Teachers at DCCs were trained to distribute questionnaires to parents of the children and to collect the completed surveys. Researchers then collected the completed surveys from designated teachers and entered them into a database for further analysis.

### Instruments and Procedures

The questionnaires used were adapted from the International Study on Asthma and Allergy in Children for Parents, which was validated in the Malay language by Norliza et al. [14]. The questionnaire was categorized into four sections:

- General background: gender, age, birth height and weight, breastfeeding duration, family history of asthma and allergies, etc.;
- Health outcomes: wheeze and/or night cough, nebulizer treatment, hospital admission, and physician-diagnosed asthma or allergies within the previous 12 months;
- Home characteristics: location of home, type of residence, size of residence, near main road/industrial area, presence of pets;
- Other factors: breastfeeding, total duration of daycare attendance, age first entering DCC, environmental tobacco smoke exposure, vehicle used to go to DCCs.

Several air-monitoring instruments were used to conduct IAQ assessment in this study. Physical, chemical, and biological parameters were assessed. For physical parameters, temperature, relative humidity, air velocity, and particulate matter PM2.5 and PM10 were measured; for chemical parameters, carbon monoxide (CO) and carbon dioxide (CO_2_) were measured; and for biological parameters, total bacterial count (TBC) and total fungus count (TFC) were measured. The monitoring instruments used were a TSI 8520 DustTrak Airborne Particle Monitor for PM2.5 and PM10; Q-Trak Plus Model 8554 Monitor for CO_2_, CO, relative humidity, and temperature; and TSI Velocicalc Plus Model 8386 for air velocity. The instruments were placed at the height about 0.6–1.5 m above the floor, near the level of the children’s breathing zone. The instruments were placed in an area not closer than 1 m to a wall, door, window, or active heating system. Instruments for particulate matter assessment were used based on real-time monitoring while the measurements of CO_2_, CO, temperature, relative humidity, and air velocity were taken periodically and spread throughout many areas in the building so that the coverage was distributed evenly. The indoor air parameters were measured in the playroom at a time when most of the children occupied the room. The children are in the playroom most of the time in almost all DCCs; the playroom was also used for children to take their nap and rest. The outdoor air parameters were measured concurrently with the indoor air parameters. The time of measurement was from 8:00 AM to 5:00 PM, during the DCC’s operation hours.

For biological sampling, the Anderson principle was used and the Merck Mas-100 Eco, a microbial air sampler with the air volume of 0.200 m^3^, was used as the sampling device. Before sampling, the inside area of the sampler was disinfected with 70% alcohol and then inserted with a media plate. The media plates used were tryptic soy agar for bacterial isolation with cycloheximide 500 mg to suppress the growth of fungi and malt extract agar (MEA 2%) for fungal isolation, with chloramphenicol added to inhibit bacterial growth. The sampling time was 10 minutes [15]. After the sampling was completed, plates were removed and incubated at 37°C for 1–2 days for bacterial culture and at 22°C–25°C for five days for fungi culture [4]. After incubation, the bacterial and fungal colonies were counted and calculated to express colony forming unit/m^3^ by the following formula:

\begin{array}{l}
{\rm{Total\ counts}} ({\rm{colony}} - {\rm{forming\ unit}}[{\rm{CFU}}]/{{\rm{m}}^3})\\
\qquad \qquad \qquad = [{\rm{Total\ colonies}} \times 1,000]/200{\kern 1pt}
\end{array}

All the samples were taken during the hot season, with minimal change in temperature and weather within the six-month period (January to June, 2014), and no renovations or painting activities were carried out at any DCC during the sampling.

### Data Analysis

Statistical analysis was carried out using SPSS version 21.0. To determine the predictors of wheezing symptom among children; simple and multiple logistics regressions were used. A *P* value < 0.05 was considered significant for all analyses.

## Results

### Sociodemographic Characteristics of Respondents

The results in Table 1 show the characteristics of respondents from the 10 DCCs investigated. The majority of them were from middle-income families and lived in terrace-type houses. Most of them had early exposure to DCC, starting at less than one year of age, and had an average duration of stay in the DCC of 1–3 years. Less than 20% of the respondents had family history of asthma or food allergy; however, most of them were exposed to environmental tobacco smoke at home. Most parents had a high education level—bachelor’s, master’s, or doctorate level.

**Table 1:** Sociodemographic Characteristics of Respondents and Wheezing Symptoms (n = 90).

Variables	f (%) or mean (SD)	Wheezing Symptom		*X*^2^/t	*P*

		Yes (n = 17) f (%) or Mean (SD)	No (n = 73) f (%) or Mean (SD)		

Age*	1.54 (0.51)	1.35 (0.50)	1.59 (0.49)	1.772	0.080
Sex					
Male	50 (55.6)	12 (24.0)	38 (76.0)	1.918	0.166
Female	40 (44.4)	5 (12.5)	35 (87.5)		
Birth weight (kg)^†^	3.05 (0.39)	3.04 (0.39)	3.05 (0.40)	0.146	0.884
Breastfeeding					
Yes	86 (45.6)	16 (18.6)	70 (81.4)	0.102	0.749
No	4 (4.4)	1 (25.0)	3 (75.0)		
Duration of breastfeeding					
Less than 6 mo.	33 (36.7)	4 (12.1)	29 (87.9)	1.558	0.212
6 mo. and above	57 (63.3)	13 (22.8)	44 (77.2)		
Food allergy					
Yes	10 (11.1)	4 (40.0)	6 (60.0)	3.273	0.070
No	80 (88.9)	13 (16.3)	67 (83.8)		
ETS exposure at home					
Yes	50 (55.6)	9 (18.0)	41 (82.0)	0.058	0.810
No	40 (44.4)	8 (20.0)	32 (80.0)		
Age entering DCC					
Less than 1 y	34 (37.8)	10 (29.4)	24 (70.6)	6.852	0.037^†^
1–2 y	29 (32.2)	6 (20.7)	23 (79.3)		
More than 2 y	27 (30.0)	1 (3.7)	26 (96.3)		
Duration of stay in DCC					
More than 3 y	8 (8.9)	1 (12.5)	7 (87.5)	4.565	0.012^†^
1–3 y	48 (53.3)	13 (27.1)	35 (72.9)		
Less than 1 y	34 (37.8)	3 (8.8)	31 (91.2)		
Household income					
Low income	18 (20.0)	2 (11.1)	16 (88.9)	21.194	<0.001^†^
Middle income	67 (74.4)	14 (20.9)	53 (70.1)		
High income	5 (5.6)	1 (20.0)	4 (80.0)		
Parent’s level of education					
High education	64 (71.1)	0 (0)	26 (100.0)	8.515	0.004^†^
Low education	26 (28.9)	17 (26.6)	47 (73.4)		
Family history of asthma					
Yes	19 (21.1)	4 (21.1)	15 (78.9)	0.074	0.786
No	71 (78.9)	13 (18.3)	58 (81.7)		
Type of housing					
Detached house	9 (10.0)	0 (0)	9 (100.0)	2.373	0.305
Terraced	77 (85.6)	16 (20.8)	61 (79.2)		
Apartments/condominium/flats	4 (4.4)	1 (25.0)	3 (75.0)		
Residence near major roadway					
Yes	40 (44.4)	5 (12.5)	35 (87.5)	1.918	0.166
No	50 (55.6)	12 (24.0)	38 (76.0)		
Pets at home					
Yes	19 (21.1)	3 (15.8)	16 (84.2)	0.151	0.698
No	71 (78.9)	14 (19.7)	57 (80.3)		
Carpets at home					
Yes	44 (48.9)	10 (22.7)	34 (77.3)	0.828	0.363
No	46 (51.1)	7 (15.2)	39 (84.8)		

DCC = day care center; ETS = environmental tobacco smoke; SD = standard deviation. *Mean (SD). ^†^ Significant level at *P* value < 0.05.

From the results, there was a significant association between parental education level and wheezing symptoms (X^2^ = 8.515, *P* = 0.004). Age the children first entered the DCC, duration of day care attendance, and household income were also significantly associated with wheezing symptoms (X^2^ = 6.852, *P* = 0.037; X^2^ = 4.565, *P* = 0.012; X^2^ = 21.194, *P* < 0.001). However, type of house, pets at home, and residences located near major road had no significant association with wheezing symptoms.

### Characteristics of DCCs

A majority of the DCC buildings were more than 10 years old, as tabulated in Table 2. Nine of the DCCs were located in residential buildings while one was located in a commercial building. Seven of the DCCs were run by private agencies and three were run by the government. All DCCs were located in an urban area in the Seremban district. Mechanical ventilation was used by each of the selected DCCs. All centers were cleaned daily by mopping and/or vacuuming.

**Table 2:** Sampling Sites and Environmental Conditions of 10 Air-Conditioned DCCs in Seremban District, Negeri Sembilan.

DCC	Building Age (y)	Number of Occupants (2014/2015)	Type of Building		Location	Site	Temp (°C) Mean (SD)	R.H. (%) Mean (SD)

A	25+	16	Private residential	Urban		Playroom	28.40 (0.1)	71.12 (0.4)
B	15+	20	Private residential	Urban	*Near roadway	Playroom	28.58 (0.2)	65.63 (0.8)
C	10+	14	Private residential	Urban	*Near industrial area (<1 km)	Playroom	31.18 (0.1)	59.60 (0.5)
D	25+	32	Public residential	Urban		Playroom	28.81 (0.3)	67.00 (0.3)
E	25+	28	Public residential	Urban		Playroom	28.58 (0.2)	72.78 (0.7)
F	5+	25	Private residential	Urban		Playroom	30.67 (0.1)	74.30 (0.4)
G	10+	10	Private residential	Urban		Playroom	28.10 (0.2)	69.8 (0.5)
H	10+	30	Private residential	Urban	*Near construction site	Playroom	27.30 (0.4)	67.10 (0.4)
I	5+	19	Commercial building	Urban	*Near roadway	Playroom	27.17 (0.2)	79.00 (0.7)
J	10+	17	Private residential	Urban	*Near roadway	Playroom	27.54 (0.1)	78.00 (0.6)

DCC = day care center; R.H. = relative humidity; SD = standard deviation; Temp = temperature. *Indicates that center near to pollution source.

### Prevalence of Wheezing Symptoms

The symptom assessed in this study was wheezing, identified using the adapted and validated questionnaires from the International Study on Asthma and Allergy in Children. The questions discussed wheezing, night cough, child waking up from sleep due to symptoms, child ever received any nebulizer treatment, admittance to ward, and diagnosis of asthma by physicians. Table 3 shows the prevalence of health symptoms among children from each DCC.

**Table 3:** Prevalence of Health Symptoms and Severity of Symptoms Among Respondents in 10 DCCs (n = 90).

DCC, N = 90	Wheezing Only, n (%)	Night Cough Only, n (%)	Both Symptoms (Wheezing and Night Cough), n (%)	Wake up from Sleep, n (%)	Nebulizer Treatment, n (%)	Admit to Ward, n (%)	Doctor Diagnosed Risk of Asthma, n (%)

A n = 6	3 (50.0)	5 (83.3)	2 (33.3)	3 (50.0)	2 (33.3)	0 (0)	1 (16.7)
B n = 14	12 (78.6)	5 (35.7)	5 (35.7)	9 (64.3)	5 (35.7)	1 (7.1)	3 (21.4)
C n = 7	4 (57.1)	7 (100.0)	4 (57.1)	6 (85.7)	3 (42.9)	2 (28.6)	2 (28.6)
D n = 6	1 (16.7)	4 (66.7)	1 (16.7)	0 (0)	1 (16.7)	1 (16.7)	1 (16.7)
E n = 7	7 (100.0)	4 (57.1)	0 (0)	2 (28.6)	0 (0)	0 (0)	1 (14.3)
F n = 11	11 (100.0)	7 (63.6)	0 (0)	1 (9.1)	0 (0)	0 (0)	1 (9.1)
G n = 4	4 (100.0)	2 (50.0)	0 (0)	2 (50.0)	1 (25.0)	1 (25.0)	1 (25.0)
H n = 15	1 (6.7)	10 (66.7)	1 (6.7)	4 (26.7)	0 (0)	0 (0)	1 (6.7)
I n = 10	1 (10.0)	2 (20.0)	1 (10.0)	1 (10.0)	1 (10.0)	1 (10.0)	1 (10.0)
J n = 10	2 (20.0)	6 (60.0)	1 (10.0)	3 (30.0)	2 (20.0)	1 (10.0)	3 (30.0)

DCC = day care center.

### IAQ Parameters

A total of 160 air samples were collected for assessment of bacterial and fungal counts, and 30 samples were collected to assess the PM2.5 and PM10 levels (Table 4). In this study, it was found that the mean PM2.5 level was higher in the bedroom area (113.21 ± 103.52) compared with that in the playroom area (69.35 ± 47.61). The indoor PM10 level was not much different from the outdoor PM10 level (81.11 ± 45.29 vs. 79.61 ± 43.69). For bacterial and fungal counts, it was found that the highest mean for both were inside the bedroom area (754.80 ± 326.22 and 699.68 ± 385.61) compared with that in the playroom area (566.63 ± 359.03 and 643.25 ± 312.09). For bacterial counts, the indoor mean level was three times higher than outdoor mean levels, whereas for fungal counts, there was no difference between the indoor and outdoor levels (Table 5).

**Table 4:** Sampling Point and Number of Air Samples of the Investigated DCCs.

Sampling Points	No. of Rooms	No. of Air Samples for PM2.5 and PM10 Level	No. of Air Samples for Microbial Count (TBC and TFC)

Playroom	10	10	60
Bedroom	10	10	60
Outdoor (outside the building)	–	10	40

DCC = day care center; No. = number; PM = particulate matter; TBC = total bacterial count; TFC = total fungus count.

**Table 5:** Mean and Standard Deviation of IAQ Parameters from the Investigated DCCs.

Rooms/Area	PM2.5 Level (μg/m^3^)	PM10 Level (μg/m^3^)	TBC (CFU/m^3^)	TFC (CFU/m^3^)

Playroom				

Mean ± SD	69.35 ± 47.61	81.11 ± 45.29	566.63 ± 359.03	643.25 ± 312.09
Min–Max	17–170	28–144	155–1310	268–1182
Bedroom				

Mean ± SD	113.21 ± 103.52	79.91 ± 46.42	754.80 ± 326.22	699.68 ± 385.61
Min–Max	30–430	30–170	229–1279	269–1489
Outdoor				

Mean ± SD	70.11 ± 49.23	79.61 ± 43.69	204.21 ± 123.11	568.42 ± 313.17
Min–Max	39–540	29–350	178–980	200–560

DCC = day care center; IAQ = indoor air quality; ICOP = Indoor Air Quality Code of Practice; NAAQ = National Ambient Air Quality; PM = particulate matter; SD = standard deviation; TBC = total bacterial count; TFC = total fungus count.

**Figure 2:**
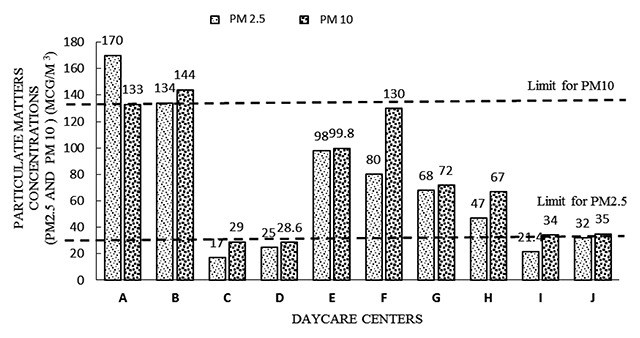
Comparison of PM2.5 and PM10 levels in each of the DCCs investigated. Abbreviations: CFU = colony-forming unit; DCC = day care center; PM = particulate matter.

**Figure 3:**
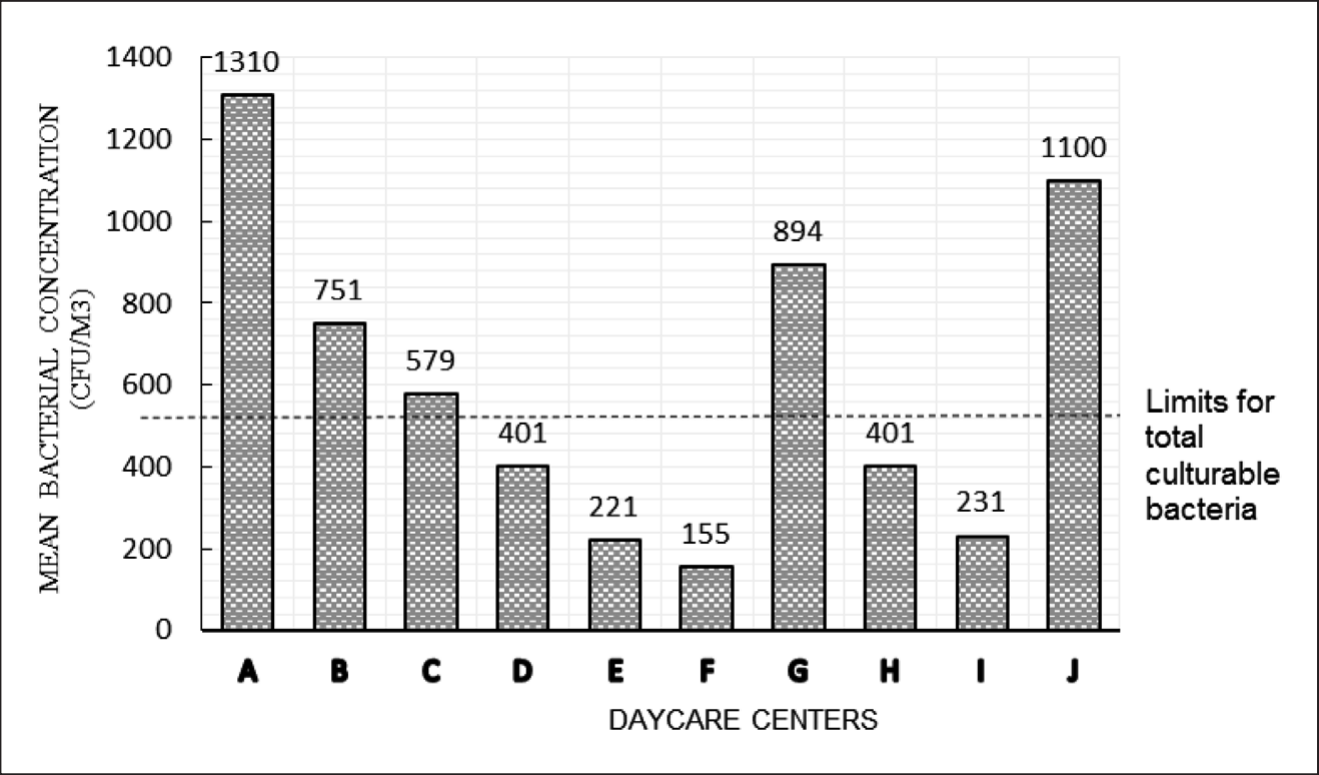
Comparison of TBC levels in each of the DCCs investigated. Abbreviations: CFU = colony-forming unit; DCC = day care center; TBC = total bacterial count.

**Figure 4:**
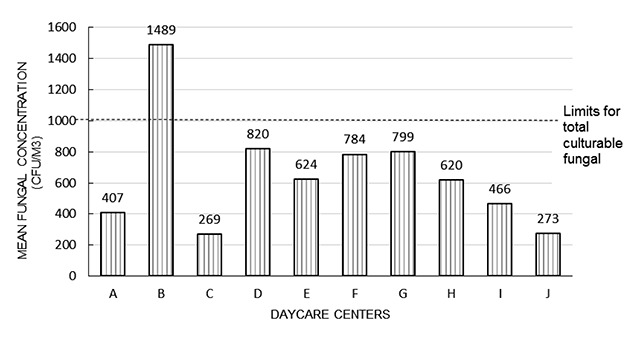
Comparison of TFC levels in each of the DCCs investigated. Abbreviations: CFU = colony-forming unit; DCC = day care center; TFC = total fungus count.

The indoor air levels were compared with the IAQ guidelines from the National Institute of Occupational Safety and Health, Malaysia [16] for bacterial count, fungal count, and PM10 levels, whereas PM2.5 levels were compared with the National Ambient Air Quality guidelines from Environmental Protection Agency (United States) or USEPA 2010, since the standard guideline for PM2.5 is not yet available in Malaysia. It was found that almost all DCCs had at least one IAQ parameter that exceeded the acceptable levels. The results in Table 6 show that DCC B had three IAQ parameters exceeding the acceptable levels, namely PM2.5, TBC, and TFC. Most DCCs had PM2.5 levels above the standard guideline, and half of the DCCs had bacterial counts exceeding acceptable levels. The bar graphs in Figures 2, 3 and 4 show IAQ parameters in each DCC that exceed the acceptable levels. The NIOSH has set 1000 CFU/m^3^ as an acceptable level for airborne bacteria and 500 CFU/m^3^ for culturable count of total bacteria. The acceptable level for PM10 is set as 150 μg/m^3^ while, for PM2.5, the acceptable level is set at 35 μg/m^3^ by the National Ambient Air Quality Guideline [17].

**Table 6:** Mean Concentrations of Indoor Air Quality Parameters at 10 Air-Conditioned DCCs Investigated in the District of Seremban, Negeri Sembilan.

DCC	Carbon Dioxide (ppm)*	PM2.5 (μg/m^3^)^†^	PM10 (μg/m^3^)*	Airborne Bacteria (CFU/m^3^)*	Airborne Fungi (CFU/m^3^)*

A	578.38	170.00^‡^	133.00	1310^‡^	514
B	669.25	134.00^‡^	144.00	751^‡^	1182^‡^
C	527.50	17.00	29.00	579^‡^	268
D	474.00	25.00	28.60	401	1161^‡^
E	519.78	98.00^‡^	99.80	221	662
F	480.89	80.00^‡^	130.00	155	460
G	848.00	68.00^‡^	72.00	894^‡^	549
H	1322.00^‡^	47.00^‡^	67.00	401	634
I	427.38	21.40	34.00	231	534
J	621.86	32.00	35.00	1100^‡^	268

CFU = colony-forming unit; DCC = day care center; PM = particulate matter. * ICOP [17]. ^†^ NAAQ [18]. ^‡^ Exceeded the acceptable level.

### Association Between IAQ Parameters and Wheezing

The association between IAQ parameters in DCCs and the prevalence of wheezing was established using the mean value. A significant association was observed between wheezing and indoor PM2.5 concentration in DCCs (*P* = 0.050, 95% CI = –37.71, –0.19), whereas no significant association was found with indoor PM10 (Table 7). For indoor bioaerosols, there was a significant association between wheezing and indoor TBC in DCCs (*P* = 0.020, 95% CI = –341.38, –30.41); however, no significant association was found with indoor TFC. No significant association was noted between wheezing and other IAQ parameters, namely carbon dioxide, temperature, and relative humidity. There was also no significant association between the DCC status (acceptable IAQ vs. unacceptable IAQ) and the symptom of wheezing.

**Table 7:** Association Between IAQ Parameters and Wheezing Symptom Among Respondents in 10 DCCs Investigated.

IAQ Parameters	Wheezing Symptom		*t* Statistic*	df	*P* value	95%CI	
	
	Yes (n = 17) Mean (SD)	No (n = 73) Mean (SD)				Upper	Lower

Carbon dioxide (ppm)	626.97 (193.96)	703.57 (320.17)	0.945	88	0.347	–84.52	237.71
Particulate matter 2.5 (μgm^3^)	82.67 (64.61)	66.25 (42.68)	–1.285	88	0.020^†^	–41.71	–0.39
Particulate matter 10 (μgm^3^)	84.38 (54.75)	80.35 (43.21)	–0.329	88	0.743	–28.40	20.32
Total bacterial count (CFU/m^3^)	778.65 (335.63)	517.26 (348.17)	–2.806	88	0.006^†^	–446.51	–76.25
Total fungal count (CFU/m^3^)	670.05 (404.31)	637.01 (290.38)	–0.391	88	0.697	–201.18	135.10

CFU = colony-forming unit; CI = confidence interval; DCC = day care center; df = degrees of freedom; IAQ = indoor air quality; SD = standard deviation. * Student’s *t* test. ^†^ *P* < 0.05 is significant.

The regression model in Table 8 showed that the predictors for wheezing symptoms among toddlers were parent’s level of education, family history of asthma, and DCC status. Children whose parents had low education level had 1.79 higher odds of having wheezing symptoms (*P* = 0.035). Those with family history of asthma had 1.08 higher odds to wheeze, and if their DCC IAQ status was unacceptable, their odds for wheezing were 7.42 times higher (*P* = 0.040; *P* = 0.032).

**Table 8:** Factors Associated with Wheezing Symptoms Among Toddlers in Day Care Centers in the District of Seremban.

Variables	Simple Logistic Regression			Multiple Logistic Regression*		

	Regression Coefficient (b)	Crude OR	*P*	Regression Coefficient (b)	Adjusted OR	*P*

Age	–0.966	0.381	0.085			
Sex						
Male	0.793	2.211	0.173			
Female	1.00					
Birth weight (kg)	–0.103	0.902	0.883			
Breastfeeding						
Yes	1.00					
No	0.377	1.458	0.749			
Duration of breastfeeding						
Less than 6 mo	–0.762	0.467	0.219			
6 mo and greater	1.00					
Food allergy						
Yes	–1.234	0.291	0.083			
No	1.00					
ETS exposure at home						
Yes	0.130	1.139	0.810			
No	1.00					
Age enter DCC						
Less than 1 y	1.914	6.783	0.087			
1–2 y	2.383	10.833	0.037^†^			
More than 2 y	1.00					
Duration of stay in DCC						
More than 3 y	–0.389	1.677	0.015^†^			
1–3 y	0.956	2.600	0.392			
Less than 1 y	1.00					
Household income						
Low income	–0.693	0.500	0.607			
Middle income	0.055	1.057	<0.001^†^			
High income	1.00					
Parent’s level of education						
High education	1.00					
Low education	–20.186	2.890	0.004^†^	–56.090	1.790	0.035^†^
Family history of asthma						
Yes	–0.174	0.841	0.786	–2.439	1.087	0.040^†^
No	1.00					
Type of housing						
Detached house	–20.104	0.051	0.380			
Terraced	–0.240	0.787	0.840			
Apartment/condominium/flat	1.00					
Residence near major roadway						
Yes	0.793	2.211	0.173			
No	1.00					
Pets at home						
Yes	0.270	1.310	0.698			
No	1.00					
Carpets at home						
Yes	0.494	1.639	0.366			
No	1.00					
DCC Status						
Acceptable	1.00					
Not acceptable	–1.153	2.316	0.040^†^	2.004	7.417	0.032^†^

Multicollinearity and interaction term was checked and not found. Hosmer-Lemeshow test, (*P* = 0.300). Classification table (overly correctly classified percentage = (69.3%). Ngelkerke R^2^ = 0.28. DCC = day care center; ETS = environmental tobacco smoke; LR = logistic regression; OR = odds ratio. * Forward LR multiple logistic regression model was applied. ^†^ Significant level at *p* < 0.05.

## Discussion

IAQ parameters were measured in 10 DCCs in this study. DCC B had the highest concentrations of PM2.5 (174 μg/m^3^), TBC (751 CFU/m^3^), and TFC (1182 CFU/m^3^). It was found that the DCC B building was more than 10 years old; it was run by a private agency and located near a major roadway. The prevalence of wheezing symptoms among the respondents was also the highest in DCC B, with 12 of 14 respondents reporting symptoms. The major contributors to the high level of indoor PM2.5 and PM10 were the indoor and outdoor combustion activity as well as the location of the DCCs. Some of the DCCs in private residential buildings had a kitchen in the building. Cooking was done inside the building, which might have led to higher concentrations of PM2.5 in these DCCs. According to Kamens et al. [18], indoor cooking can generate particles having a diameter <0.1 μg/m^3^, which accounted for 30% of the particle’s volume. Previous studies have shown that the outdoor particle pollution related to road traffic can cause ambient particulate pollution, especially fine particles, in buildings located near major roadways [19,20]. Dust generated from paved or unpaved roads as outdoor sources might also contribute to high levels of indoor particulate matter. A study in Bangkok, Thailand, revealed that indoor particulate matter concentration, mainly PM2.5, appeared to be high in buildings located in urban areas or close to motorways [21]. A study by Chua et al. [22] at a preschool also found high concentration of PM2.5 (112.62 ± 32.82 μg/m^3^) in an urban area.

The levels of indoor particulate matter and microbial count depend on several variables such as temperature, relative humidity, air movement, number of occupants, activities, and ventilation [23]. The concentration of particulate matter or dust was associated with microbial level because dust acts as medium for microbial growth [24]. This explains why some of the DCCs with high bacterial and fungal counts also had particulate matter concentrations exceeding the acceptable level.

This study found that total bacterial counts exceeded acceptable levels in half of the DCCs surveyed. Human activities such as cleaning and cooking, as well as children’s activities, could be a major source of bacteria in DCCs [25]. In this study, it was found that the total bacterial mean concentration was 566.63 ± 359.03 CFU/m^3^ in playroom areas and 754.80 ± 326.22 CFU/m^3^ in bedroom areas. A Korean study of 10 DCCs revealed that indoor total bacterial count ranged from 645.3 ± 49.0 CFU/m^3^ to 898.5 ± 86.5 CFU/m^3^, slightly higher than in our study. In Malaysia, the recommended level established by NIOSH for the culture count of total bacteria should not exceed 500 CFU/m^3^ [17]. However, it is impossible to propose an exposure limit to pathogens because information on the dose-response relationship and epidemiological data are not sufficient [26].

Children, especially toddlers, are particularly sensitive to air pollution from various sources such as dust, particulate matter, bioaerosols, etc. [24]; it can increase susceptibility of children to respiratory infections and aggravate health problems related to heart and lung diseases such as asthma [27]. The prevalence of wheezing among the respondents in this study was 18.9%. Studies done at DCCs in urban areas of Kuala Lumpur and Selangor showed the prevalence of 11.4% for wheezing and 36.2% for cough in Kuala Lumpur [22] and 32.8% for wheezing and 34.4% for night cough in Selangor [28]. This study revealed a significant association between wheezing and indoor PM2.5 level as well as TBC. A study by Nazariah et al. [29] showed a significant association of indoor fine particles with cough (OR = 1.81, CI 95% = 1.18–2.79) and wheezing (OR = 5.43, CI 95% = 2.21–13.37). A study by Mohd Nor Rawi et al. at preschools also revealed significant association between wheezing symptoms and indoor PM2.5 [28]. The long-term effects of PM2.5 particles on children include lung function changes and development of chronic respiratory disease. A study by Sonnenschein-van der Voort et al. [30] suggests that long-term exposure to higher levels of traffic-related air pollutants such as PM2.5 was associated with increased risk of wheezing in the first three years of life. In another study, there was an association between indoor PM2.5 and overall wheezing until eight years of age [31].

In DCCs, children’s activities such as talking, sneezing, coughing, walking, washing, and toilet flushing can generate airborne biological particulate matter [25]. Biological contaminants such as bacteria and fungi can trigger allergic reaction, including hyperactive airway disease, allergic rhinitis, and asthma [27]. Bacteria are believed to have harmful effects due to endotoxin production, which can induce inflammation of the airways, increase bronchial hyperactivity, and elicit asthmatic attacks, and this endotoxin is normally shed by bacteria found in household dust [32]. In this study, there was a significant association between asthmatic symptoms and bacterial count concentration (*P* = 0.020, 95% CI = –341.38, –30.41). A study in Bangkok showed that domestic endotoxin levels were associated with the frequency of wheezing episodes in asthmatic children [21], and a study of indoor air microbes and respiratory symptoms among school children showed a significant association between endotoxin levels and night cough symptoms, with a prevalence of 16% [33]. Despite this, some researchers believe that microbial exposure in early life might protect children from developing atopy and allergic asthma; however, this mechanism is still not well understood [34].

In this study, all DCCs used mechanical ventilation (air conditioners). From Table 5 it can be seen that, for the playroom area, the indoor PM2.5 level was slightly lower than the outdoor level (69.35 vs. 70.11), while PM10 level was slightly higher than the outdoor level (81.11 vs. 70.11). However, indoor TBC and TFC concentrations were higher than outdoor concentrations. For the bedroom area, all of the parameters had higher mean concentrations indoors compared with those outdoors. A study in Singapore by Zuraimi et al. [35] at 104 DCCs revealed the same results whereby indoor particulate matter and bioaerosols had higher concentrations compared with those outdoors. This study also found that air-conditioned centers have significantly lower air-exchange rates than naturally ventilated centers. The air-exchange rates will affect the penetration of the outdoor pollutants [36]. Most domestic air conditioners have no fresh intake, and this could affect the IAQ of the area significantly [37]. These ventilation inadequacies in particular failed to dilute pollutants generated by the occupants such as human-related bacterial levels, resulting in higher concentrations of pollutants indoors compared with those outdoors [36]. However, a recent study suggests that opening windows and using air purifiers may help to reduce the indoor air pollutant level [25].

## Conclusion

This study provides data regarding the levels of IAQ parameters in urban DCCs. Findings from this study indicate that exposure to poor IAQ and increasing levels of indoor air pollutants were associated with respiratory symptoms, particularly wheezing, among toddlers in DCCs. The indoor particle concentrations and presence of microbes in DCCs might increase the risk in exposed children for respiratory diseases, particularly asthma, later in life. It is suggested that DCC management, parents, and the public be educated and that a conducive environment, with safe IAQ, be developed in DCCs to protect children’s health.
